# Association of Resistance-Associated *23S rRNA* and *gyrA* Mutations with Antimicrobial Resistance and Eradication Outcomes in *Helicobacter pylori*

**DOI:** 10.3390/antibiotics15070661

**Published:** 2026-07-04

**Authors:** Sergiu Dorin Matei, Tiberia Ilias, Ramona Nicoleta Suciu, Corina Suteu, Cornel Dragos Cheregi, Laura Ioana Bondar, Anamaria Violeta Țuțuianu, Brigitte Osser, Ovidiu Frățilă

**Affiliations:** 1 Doctoral School of Biomedical Sciences, Faculty of Medicine and Pharmacy, University of Oradea, 410087 Oradea, Romania; sdmatei@uoradea.ro (S.D.M.); brigitte.osser@uav.ro (B.O.); ofratila@uoradea.ro (O.F.); 2 Department of Medical Disciplines, Faculty of Medicine and Pharmacy, University of Oradea, 410073 Oradea, Romania; 3 Department of Psycho-Neuroscience and Recovery, Faculty of Medicine and Pharmacy, University of Oradea, 410073 Oradea, Romania; corina.suteu@didactic.uoradea.ro; 4 Department of Surgical Disciplines, Faculty of Medicine and Pharmacy, University of Oradea, 410073 Oradea, Romania; cornel.cheregi@didactic.uoradea.ro; 5 Department of Biology and Life Sciences, Faculty of Medicine, “Vasile Goldiș” Western University of Arad, 310025 Arad, Romania; bondar.laura@uvvg.ro; 6 Department of Dentistry, Faculty of Medicine and Pharmacy, University of Oradea, 410073 Oradea, Romania; anamaria.tutuianu@uoradea.ro; 7 Faculty of Physical Education and Sport, “Aurel Vlaicu” University of Arad, 310130 Arad, Romania

**Keywords:** antimicrobial resistance, clarithromycin resistance, eradication failure, fluoroquinolone resistance, *Helicobacter pylori*, molecular diagnostics, personalized medicine, prediction model, resistance-associated mutations, susceptibility-guided therapy

## Abstract

**Background/Objectives**: The increasing prevalence of antimicrobial resistance has become a major challenge in the management of *Helicobacter pylori* infection and is a leading cause of eradication failure. Resistance to clarithromycin and fluoroquinolones is primarily mediated by mutations in the *23S rRNA* and *gyrA* genes, respectively. This study aimed to evaluate the prevalence of resistance-associated mutations in the *23S rRNA* and *gyrA* genes, investigate their relationship with phenotypic antimicrobial resistance, assess their impact on eradication outcomes, and develop a prediction model for treatment failure. **Methods**: This retrospective real-world cohort study included 294 adult patients with confirmed *H. pylori* infection evaluated at the Oradea County Emergency Clinical Hospital, Romania, between November 2022 and November 2025. Clinical, endoscopic, histopathological, microbiological, molecular, and treatment outcome data were collected from medical records. Resistance-associated mutations in the *23S rRNA* (A2143G, A2142G, and A2142C) and *gyrA* (N87K, D91G, and D91N) genes were analyzed and correlated with phenotypic antimicrobial resistance and eradication outcomes. Independent predictors of eradication failure were identified using multivariable logistic regression, and a prediction model was subsequently developed. **Results**: Overall, 101 patients (34.4%) harbored *23S rRNA* mutations and 64 (21.8%) carried *gyrA* mutations, while 27 patients (9.2%) exhibited mutations in both genes. A2143G was the most frequent mutation (25.2%). Resistance-associated mutations showed strong concordance with phenotypic antimicrobial resistance. Patients with wild-type strains achieved eradication rates exceeding 90%, whereas significantly lower success rates were observed among patients carrying A2143G, A2142G, or *gyrA* mutations. Multivariable analysis identified previous eradication attempts (aOR 3.12, 95% CI 1.71–5.68), A2143G mutation (aOR 4.86, 95% CI 2.43–9.72), *gyrA* mutation (aOR 2.91, 95% CI 1.45–5.84), increasing age (aOR 1.03, 95% CI 1.01–1.05), and treatment with clarithromycin-based triple therapy (aOR 2.18, 95% CI 1.02–4.63) as independent predictors of eradication failure. The prediction model demonstrated excellent discriminatory performance (AUC 0.88, 95% CI 0.84–0.92), with a sensitivity of 82.5%, specificity of 80.1%, and satisfactory calibration (Hosmer–Lemeshow *p* = 0.68). **Conclusions**: Resistance-associated mutations in the *23S rRNA* and *gyrA* genes are strongly associated with phenotypic antimicrobial resistance and reduced *H. pylori* eradication success. Molecular resistance testing may facilitate individualized treatment selection and improve clinical outcomes. The proposed prediction model, integrating clinical characteristics, treatment regimen, and molecular resistance markers, demonstrated excellent performance and may represent a useful tool for identifying patients at increased risk of eradication failure.

## 1. Introduction

*Helicobacter pylori* is one of the most prevalent chronic bacterial infections worldwide, affecting approximately half of the global population. The infection is recognized as the primary etiological factor for chronic gastritis, peptic ulcer disease, gastric mucosa-associated lymphoid tissue lymphoma, and gastric adenocarcinoma. Consequently, the World Health Organization has classified *H. pylori* as a class I carcinogen, highlighting its major public health significance [1,2,3,4].

Successful eradication of *H. pylori* reduces the risk of peptic ulcer recurrence and gastric cancer development. However, the effectiveness of standard eradication therapies has progressively declined over recent decades, primarily due to the increasing prevalence of antimicrobial resistance [5,6,7,8]. Resistance to clarithromycin and fluoroquinolones has emerged as a major challenge worldwide and is currently considered one of the principal causes of treatment failure [9,10]. As a result, international guidelines increasingly recommend susceptibility-guided treatment strategies whenever feasible [11,12,13].

At the molecular level, resistance to clarithromycin is predominantly associated with point mutations within domain V of the *23S rRNA* gene, particularly the A2143G, A2142G, and A2142C substitutions, which impair antibiotic binding to the bacterial ribosome [14,15]. Similarly, resistance to fluoroquinolones is mainly mediated by mutations within the quinolone resistance-determining region of the *gyrA* gene, most commonly involving amino acid substitutions at positions N87 and D91 [6,10]. Numerous studies have demonstrated a strong correlation between these genetic alterations and phenotypic antimicrobial resistance, supporting their utility as molecular markers for predicting treatment response [5,16].

Despite the growing implementation of molecular resistance testing, important gaps in knowledge remain. Most available studies have focused either on the prevalence of resistance-associated mutations or on their association with antimicrobial susceptibility profiles. Comparatively few investigations have simultaneously evaluated molecular resistance markers, phenotypic resistance patterns, and eradication outcomes within the same real-world patient cohort. Furthermore, limited data are available regarding the combined predictive value of *23S rRNA* and *gyrA* mutations for identifying patients at increased risk of eradication failure and supporting individualized treatment selection.

Given the increasing burden of antimicrobial resistance and the need for precision-based management strategies, the identification of clinically relevant molecular predictors of treatment failure represents an important research priority. We hypothesized that resistance-associated mutations in the *23S rRNA* and *gyrA* genes are significantly associated with both phenotypic antimicrobial resistance and eradication failure and that these molecular markers can contribute to the development of a clinically useful prediction model.

Therefore, the aim of the present study was to evaluate the prevalence of resistance-associated mutations in the *23S rRNA* and *gyrA* genes, investigate their relationship with phenotypic antimicrobial resistance, assess their impact on *H. pylori* eradication outcomes, and develop a prediction model for identifying patients at increased risk of treatment failure in a real-world cohort from northwestern Romania.

## 2. Results

### 2.1. Clinical Characteristics

The baseline characteristics of the study population are summarized in Table 1. The cohort included 294 patients with confirmed *H. pylori* infection, with a mean age of 53.6 ± 14.2 years, and females accounted for 58.8% of the study population. Most patients resided in urban areas (71.8%). Previous eradication attempts were documented in approximately one-quarter of patients, while nearly one-third were active smokers. Dyspepsia was the most common presenting symptom. Hypertension, dyslipidemia, and diabetes mellitus represented the most frequent comorbid conditions. Additional demographic and clinical characteristics are presented in Table 1.

### 2.2. Endoscopic and Histopathological Characteristics

The endoscopic and histopathological characteristics of the study population are presented in Table 2. Antral gastritis was the most common endoscopic finding, being observed in nearly half of the patients, followed by pangastritis and erosive gastritis. Peptic ulcer disease was less frequently encountered, with gastric ulcers occurring more often than duodenal ulcers.

Histopathological evaluation revealed predominantly inflammatory gastric lesions. Intestinal metaplasia was identified in 11.2% of patients, while gastric atrophy was observed in 7.1%. Moderate gastritis represented the most frequent histological pattern. Overall, the findings indicate that inflammatory gastric pathology predominated in the studied cohort.

### 2.3. Distribution of Resistance-Associated Mutations

The distribution of resistance-associated mutations is presented in Table 3. Overall, 101 patients (34.4%) harbored at least one mutation in the *23S rRNA* gene, while 64 patients (21.8%) carried *gyrA* mutations. Concomitant *23S rRNA* and *gyrA* mutations were identified in 27 patients (9.2%). A wild-type genotype, defined as the absence of the investigated mutations, was observed in 129 patients (43.9%).

Among the *23S rRNA* mutations, A2143G was the most frequently detected variant, accounting for 25.2% of the study population. In contrast, N87K represented the predominant *gyrA* mutation, followed by D91G and D91N. Overall, mutations associated with clarithromycin resistance were more prevalent than those associated with fluoroquinolone resistance.

### 2.4. Association Between Resistance-Associated Mutations and Phenotypic Resistance

**Figure 1:**
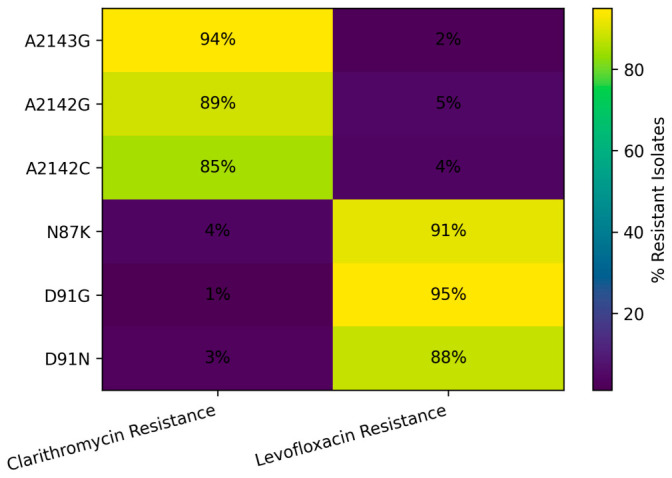
Heatmap of the Association Between Resistance-Associated Mutations and Phenotypic Resistance. Percentages represent the proportion of isolates with phenotypic clarithromycin or levofloxacin resistance among isolates harboring each resistance-associated mutation. Percentages were calculated using the number of isolates carrying the respective mutation as the denominator (A2143G, n = 74; A2142G, n = 19; A2142C, n = 8; N87K, n = 29; D91G, n = 23; D91N, n = 12). Some isolates harbored resistance-associated mutations in both the *23S rRNA* and *gyrA* genes; therefore, the mutation categories are not mutually exclusive, and the percentages should not be summed across rows.

The association between resistance-associated mutations and phenotypic antimicrobial resistance is presented in Figure 1. Mutations within the *23S rRNA* gene demonstrated a strong concordance with clarithromycin resistance, whereas *gyrA* mutations were highly associated with levofloxacin resistance.

Among the investigated variants, A2143G represented the mutation most strongly associated with clarithromycin resistance, while D91G exhibited the highest concordance with levofloxacin resistance. Cross-resistance between mutation groups and unrelated antibiotic classes was uncommon.

Overall, the observed molecular profiles showed a high degree of agreement with phenotypic susceptibility testing, supporting the utility of *23S rRNA* and *gyrA* mutations as reliable molecular markers of clarithromycin and fluoroquinolone resistance, respectively.

### 2.5. Treatment Outcomes According to Molecular Profile

Treatment outcomes according to mutation status are summarized in Table 4. Patients harboring a wild-type genotype achieved the highest eradication rates, with successful treatment observed in more than 90% of cases.

In contrast, the presence of resistance-associated mutations was significantly associated with lower eradication success. Patients carrying the A2143G mutation exhibited the lowest eradication rate and the highest frequency of treatment failure. Similarly, A2142G mutations and mutations within the *gyrA* gene were associated with significantly reduced eradication success compared with wild-type strains.

Overall, the presence of either *23S rRNA* or *gyrA* mutations was associated with a substantially increased likelihood of eradication failure, highlighting the clinical relevance of molecular resistance testing prior to treatment selection.

### 2.6. Treatment Regimens

The distribution of eradication regimens is presented in Table 5. Clarithromycin-based triple therapy was the most frequently prescribed regimen (162/294, 55.1%), followed by bismuth quadruple therapy (78/294, 26.5%), levofloxacin-based therapy (41/294, 13.9%), and other regimens (13/294, 4.4%). Patients treated with clarithromycin-based triple therapy experienced a significantly higher rate of eradication failure than those treated with bismuth quadruple therapy (34.6% vs. 9.0%, *p* < 0.001), whereas no statistically significant differences were observed for the remaining treatment regimens. Treatment regimen was therefore included as a clinically relevant covariate in the multivariable logistic regression analysis.

### 2.7. Comparison Between Successful and Failed Eradication

The clinical, microbiological, and molecular characteristics of patients according to treatment outcome are presented in Table 6. Patients who experienced eradication failure were significantly older than those who achieved successful eradication. Furthermore, previous eradication attempts were substantially more frequent among patients with treatment failure.

Phenotypic clarithromycin resistance was markedly more prevalent in the failure group. Similarly, resistance-associated molecular markers, including *gyrA* mutations and the A2143G mutation, were observed significantly more often among patients who failed eradication therapy.

No significant differences were identified regarding sex distribution, smoking status, body mass index (BMI), or urban versus rural residence (all *p* > 0.05). Collectively, these findings suggest that both antimicrobial resistance and specific molecular alterations may contribute to treatment failure and should be further evaluated in multivariable analysis.

### 2.8. Independent Predictors of Eradication Failure

**Figure 2:**
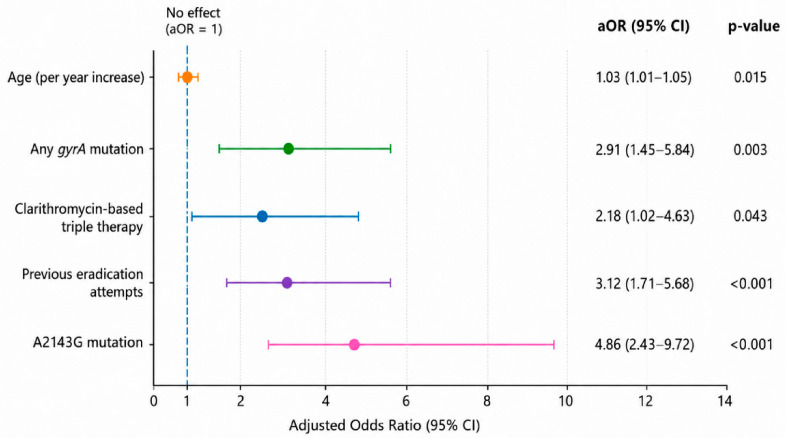
Forest Plot of Independent Predictors of Eradication Failure.

Variables demonstrating statistical significance in the univariate analysis, together with clinically relevant covariates (including eradication treatment regimen), were considered for inclusion in the multivariable logistic regression model. To minimize multicollinearity, overlapping variables representing similar biological information were not entered simultaneously into the final model. Because several variables represented overlapping measures of antimicrobial resistance (e.g., phenotypic resistance and the corresponding resistance-associated mutations), only clinically relevant, non-collinear variables were retained in the final model to minimize multicollinearity and reduce the risk of overfitting. Patients harboring concurrent *23S rRNA* and *gyrA* mutations remained in the analysis and contributed to both binary predictor variables. Therefore, the associations of these mutation groups with eradication failure were evaluated after adjustment for the other variables included in the multivariable model. Because only 27 patients harbored dual mutations, interaction terms between the two mutation groups were not included in the final model to avoid model instability. The results of the final multivariable model are presented in Table 7, while the adjusted odds ratios (aORs) are illustrated in Figure 2.

The presence of the A2143G mutation emerged as the strongest independent predictor of treatment failure, increasing the likelihood of eradication failure nearly fivefold. Previous eradication attempts were also independently associated with an increased risk of treatment failure. Similarly, *gyrA* mutations remained significantly associated with eradication failure after adjustment for potential confounders.

Increasing age was identified as an additional independent predictor, although its effect size was modest compared with molecular and treatment-related variables. Together, these findings suggest that both resistance-associated mutations and previous treatment exposure play a central role in determining eradication outcomes.

### 2.9. Development and Performance of the Prediction Model

A prediction model for *H. pylori* eradication failure was developed using the independent predictors identified in the multivariable analysis, including age, previous eradication attempts, A2143G mutation status, *gyrA* mutation status, and eradication treatment regimen.

**Figure 3:**
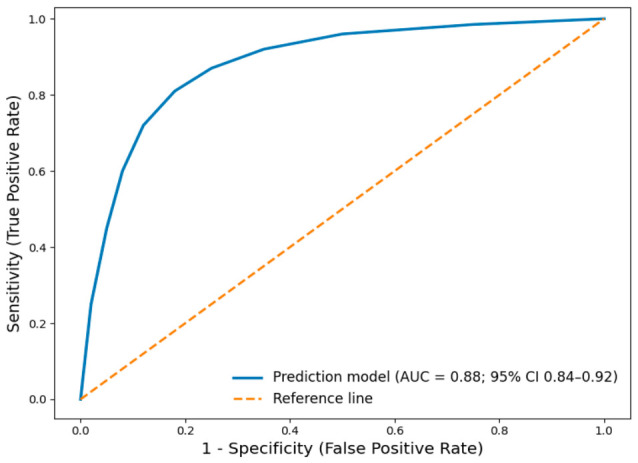
ROC Curve of the Prediction Model.

The model demonstrated good discriminatory performance, with an area under the receiver operating characteristic (ROC) curve of 0.88 (95% CI: 0.84–0.92) (Figure 3). The optimal cutoff value yielded a sensitivity of 82.5% and a specificity of 80.1% for predicting treatment failure.

**Figure 4:**
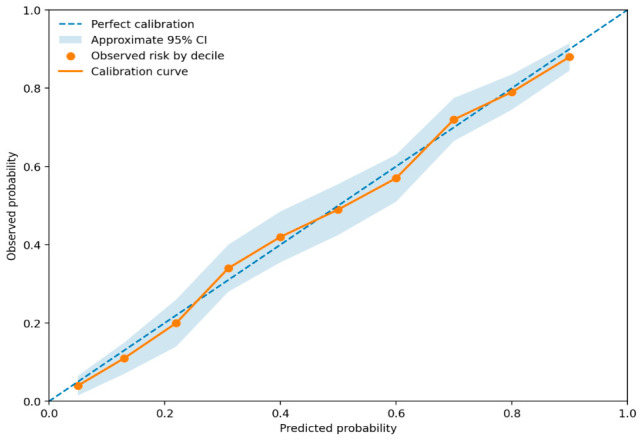
Calibration plot of the internally validated prediction model for *H. pylori* eradication failure.

Model calibration was assessed using the Hosmer–Lemeshow goodness-of-fit test. The model demonstrated satisfactory calibration, with a Hosmer–Lemeshow χ^2^ value of 5.78 and a non-significant *p*-value (*p* = 0.68), indicating good agreement between predicted and observed probabilities of eradication failure. Internal validation using bootstrap resampling (1000 iterations) demonstrated good model stability. The apparent AUC of 0.88 decreased only slightly after optimism correction, yielding a bootstrap-corrected AUC of 0.85. The calibration slope was 0.93, indicating limited overfitting and good agreement between predicted and observed probabilities of eradication failure (Figure 4).

To facilitate clinical implementation, a simplified clinical risk score was constructed by assigning points proportional to the regression coefficients of the independent predictors identified in the final multivariable model (Table 8). Patients were subsequently stratified into low-, intermediate-, and high-risk categories according to their predicted probability of eradication failure.

The probability of eradication failure increased progressively across risk categories, ranging from 7.2% in the low-risk group to 68.1% in the high-risk group (Table 9). These findings demonstrate good discriminatory performance and calibration and support the potential clinical utility of the proposed prediction model for individualized treatment selection.

## 3. Discussion

The present study investigated the prevalence and clinical significance of resistance-associated mutations in the *23S rRNA* and *gyrA* genes among patients with *H. pylori* infection and evaluated their relationship with phenotypic antimicrobial resistance, eradication outcomes, and treatment failure prediction. Several important findings emerged. First, mutations within the *23S rRNA* and *gyrA* genes were frequently detected in the study population, with A2143G representing the most prevalent clarithromycin resistance-associated mutation and N87K and D91 substitutions representing the most common fluoroquinolone resistance-associated alterations. Second, these mutations demonstrated strong concordance with phenotypic clarithromycin and levofloxacin resistance. Third, patients harboring resistance-associated mutations experienced significantly lower eradication rates than patients infected with wild-type strains. Finally, previous eradication attempts, increasing age, A2143G mutation status, *gyrA* mutations, and treatment with clarithromycin-based triple therapy emerged as independent predictors of eradication failure and were incorporated into a prediction model with excellent discriminatory performance.

Notably, patients harboring both *23S rRNA* and *gyrA* mutations exhibited the poorest treatment outcomes, whereas eradication rates exceeded 90% among patients infected with wild-type strains. Collectively, these findings support the study hypothesis and highlight the potential value of molecular resistance testing for identifying patients at increased risk of treatment failure, guiding individualized treatment selection, and improving eradication success.

### 3.1. Molecular Resistance Patterns

Mutations within the *23S rRNA* gene were identified in approximately one-third of patients, whereas *gyrA* mutations were detected in approximately one-fifth of the study population. These findings are broadly consistent with previous European and international studies reporting increasing rates of molecular resistance to both clarithromycin and fluoroquinolones [17,18,19,20].

The predominance of the A2143G mutation observed in the present study is consistent with numerous investigations identifying this substitution as the principal molecular mechanism of clarithromycin resistance in *H. pylori* [21,22,23,24]. Mutations affecting positions 2142 and 2143 within domain V of the *23S rRNA* gene alter the antibiotic-binding site of the bacterial ribosome, reducing macrolide susceptibility and contributing to treatment failure [15,25,26]. The frequency of A2143G observed in our cohort is comparable to rates reported in other European populations and further supports its role as a dominant resistance determinant [27,28,29].

Similarly, the observed prevalence of N87K, D91G, and D91N mutations within the *gyrA* gene corresponds to previous reports identifying these substitutions as the primary molecular drivers of fluoroquinolone resistance [30,31,32]. These mutations affect the quinolone resistance-determining region of deoxyribonucleic acid gyrase and reduce antibiotic binding efficiency, ultimately compromising the effectiveness of levofloxacin-containing regimens [33,34,35].

The coexistence of mutations within both genes observed in a subset of patients is particularly noteworthy. Previous studies have suggested that simultaneous resistance to clarithromycin and fluoroquinolones may represent an emerging challenge, especially in regions with widespread antibiotic exposure [36,37,38]. The prevalence of dual resistance observed in the present study emphasizes the importance of continuous resistance surveillance and individualized treatment selection.

### 3.2. Relationship Between Resistance-Associated Mutations and Phenotypic Resistance

One of the most important findings of the present study was the strong association between resistance-associated mutations and phenotypic antimicrobial resistance. Patients harboring A2143G mutations demonstrated very high rates of clarithromycin resistance, while N87K and D91G mutations showed similarly strong associations with levofloxacin resistance.

These findings are biologically plausible and consistent with current understanding of resistance mechanisms in *H. pylori* [39,40,41]. Previous investigations have demonstrated that molecular resistance markers exhibit high diagnostic accuracy for predicting phenotypic resistance and may serve as reliable alternatives to conventional culture-based susceptibility testing [35,42].

The strong concordance observed in our cohort supports the increasing use of molecular diagnostics in routine clinical practice. Compared with traditional culture methods, molecular testing offers several practical advantages, including faster turnaround times, reduced technical complexity, and the ability to detect resistance-associated mutations directly from clinical specimens [35,43,44,45].

### 3.3. Impact of Resistance-Associated Mutations on Eradication Outcomes

A particularly relevant finding of the present study was the substantial reduction in eradication success among patients harboring resistance-associated mutations. Wild-type strains achieved eradication rates exceeding 90%, whereas significantly lower success rates were observed among patients carrying A2143G, A2142G, or *gyrA* mutations.

These findings are consistent with previous studies demonstrating that antimicrobial resistance remains one of the strongest determinants of treatment failure [22,46,47,48]. Clarithromycin resistance has been repeatedly associated with markedly reduced eradication rates when clarithromycin-containing regimens are used, while fluoroquinolone resistance similarly compromises the effectiveness of levofloxacin-based therapies [5,9,49].

The poorest outcomes were observed among patients harboring mutations in both the *23S rRNA* and *gyrA* genes. This observation is particularly important because it suggests the presence of multidrug-resistant strains that may not respond adequately to standard first- or second-line treatment regimens. Similar findings have been reported in recent studies evaluating dual-resistant *H. pylori* isolates [28,50,51].

### 3.4. Clinical Significance of Molecular Resistance Markers and Other Predictors of Eradication Failure

Multivariable analysis identified previous eradication attempts, increasing age, A2143G mutation, gyrA mutation, and treatment with clarithromycin-based triple therapy as independent predictors of eradication failure. Importantly, treatment regimen was included in the multivariable model because it is a recognized determinant of *H. pylori* eradication success. After adjustment for treatment regimen, the associations between the A2143G mutation, *gyrA* mutations, and eradication failure remained statistically significant, suggesting that these molecular markers contributed to treatment failure independently of differences in the administered eradication therapies.

The association between previous eradication attempts and treatment failure is consistent with previous literature suggesting that repeated antibiotic exposure promotes the selection of resistant strains [52,53,54,55,56]. Patients who have undergone multiple eradication attempts are therefore more likely to harbor resistant organisms and experience subsequent treatment failure.

Among all evaluated predictors, A2143G mutation status demonstrated the strongest independent association with eradication failure. This finding further emphasizes the central role of clarithromycin resistance in determining treatment outcomes and supports current guideline recommendations discouraging empirical clarithromycin-based therapy in regions with elevated resistance rates [14,16,46,57,58].

The independent contribution of *gyrA* mutations is also clinically relevant. As fluoroquinolone-containing regimens are frequently used after first-line treatment failure, the increasing prevalence of *gyrA*-associated resistance may compromise the effectiveness of rescue therapies and contribute to repeated eradication failures [10,30,49,59].

### 3.5. Prediction Model Performance

An important strength of the present study was the development and internal validation of a prediction model integrating clinical variables, molecular resistance markers, and treatment regimen. The model demonstrated excellent discriminatory performance, with the area under the curve (AUC) of 0.88, indicating a high ability to distinguish between successful eradication and treatment failure.

Few previous studies have attempted to integrate molecular resistance markers into clinically applicable prediction tools [60,61,62,63]. The strong performance of our model suggests that combining readily available clinical information with molecular resistance testing may facilitate individualized risk stratification and treatment planning.

Importantly, the model also demonstrated satisfactory calibration, indicating good agreement between predicted and observed outcomes. These findings support the potential utility of the model as a decision-support tool in routine clinical practice, although external validation remains necessary.

Importantly, internal validation using bootstrap resampling demonstrated only minimal optimism and confirmed the robustness of the proposed model. Although some reduction in performance was observed after correction for overfitting, the model maintained good discriminatory ability. These findings suggest that the identified predictors provide stable prognostic information and support the potential applicability of the model in clinical practice.

### 3.6. Clinical Implications

The findings of the present study have several important implications for the clinical management of *H. pylori* infection. The strong association observed between resistance-associated mutations and both phenotypic antimicrobial resistance and eradication outcomes suggests that molecular testing may represent a valuable tool for treatment individualization in routine clinical practice.

In particular, the identification of *23S rRNA* and *gyrA* mutations before treatment initiation may facilitate the selection of antibiotic regimens with a higher probability of success. Patients harboring mutations associated with clarithromycin or fluoroquinolone resistance could potentially avoid ineffective therapies and unnecessary exposure to antibiotics that are unlikely to achieve eradication. Such an approach may reduce repeated treatment failures, limit cumulative antibiotic exposure, and contribute to antimicrobial stewardship efforts.

The results of the present study also highlight the potential clinical value of susceptibility-guided therapy. Although empirical treatment remains common in many healthcare settings, the substantial differences in eradication rates observed between wild-type and mutation-positive strains suggest that molecular resistance testing may improve treatment selection, particularly in regions with increasing resistance rates. Patients infected with wild-type strains achieved excellent eradication rates, whereas the presence of resistance-associated mutations was associated with significantly poorer outcomes. This finding supports a more personalized therapeutic approach based on resistance profiles rather than a uniform treatment strategy applied to all patients.

Another clinically relevant observation was the particularly high rate of treatment failure among patients harboring both *23S rRNA* and *gyrA* mutations. These individuals may represent a subgroup at increased risk of multidrug resistance and repeated eradication failure. Early identification of such patients could allow clinicians to consider alternative eradication regimens, susceptibility-guided treatment strategies, or closer post-treatment monitoring.

The prediction model developed in the present study may further support clinical decision-making. By integrating both molecular and clinical variables, the model demonstrated excellent ability to identify patients at increased risk of eradication failure. Risk stratification may help clinicians prioritize molecular testing, select more appropriate treatment regimens, and optimize follow-up strategies. Patients classified as high risk may benefit from individualized treatment approaches and more intensive post-treatment evaluation, whereas low-risk patients may be managed using standard therapeutic protocols.

To facilitate clinical implementation, the proposed risk score may be applied at the time of diagnosis by assigning points to each independent predictor identified in the final multivariable model. Patients accumulating higher scores are classified as having an increased probability of eradication failure and may therefore benefit from susceptibility-guided therapy, selection of bismuth-based or alternative eradication regimens, closer post-treatment follow-up, and confirmation of eradication. Conversely, patients classified as low risk may be appropriate candidates for standard first-line treatment according to current clinical guidelines. Although the proposed score requires external validation before routine clinical implementation, it provides a simple framework for individualized risk stratification and therapeutic decision-making.

Although the present study was conducted in a single tertiary referral center in Romania, the observed resistance patterns are broadly consistent with the increasing prevalence of clarithromycin and fluoroquinolone resistance reported across many European countries. Nevertheless, the prevalence of specific resistance-associated mutations and local treatment practices may vary between geographic regions. Consequently, the predictive performance of the proposed model may differ in populations with substantially different antimicrobial resistance profiles or first-line eradication strategies. External validation in multicenter cohorts from other European regions and healthcare settings is therefore warranted before widespread clinical implementation. Despite these considerations, the incorporation of well-established clinical and molecular predictors suggests that the proposed model may provide a useful framework for individualized risk stratification in settings with comparable resistance epidemiology.

Beyond antimicrobial resistance, increasing evidence indicates that the gut microbiota may also influence the effectiveness and tolerability of *H. pylori* eradication therapy. The composition of the gastrointestinal microbiota may affect host immune responses, antibiotic metabolism, treatment-related adverse events, and the likelihood of successful eradication. Furthermore, alterations in the gut microbiome induced by eradication therapy have prompted growing interest in microbiota-targeted interventions, including probiotic supplementation and personalized therapeutic approaches. Although the present study focused on resistance-associated molecular markers, future prediction models may benefit from integrating microbiome-related factors to further improve individualized treatment strategies. These observations are consistent with recent evidence highlighting the role of the gut microbiota in optimizing therapeutic regimens across gastrointestinal diseases and other clinical conditions [64,65].

Collectively, these findings support the integration of molecular resistance testing into routine *H. pylori* management and highlight its potential role in advancing precision medicine approaches within gastroenterology, particularly in regions with increasing antimicrobial resistance rates.

### 3.7. Limitations of the Study

Several limitations should nevertheless be acknowledged. First, the retrospective observational design limits the ability to establish causal relationships between resistance-associated mutations and treatment outcomes. Although strong associations were identified, residual confounding cannot be completely excluded, and prospective studies are required to confirm these findings.

Second, this was a single-center study conducted at a tertiary referral hospital in northwestern Romania. Consequently, local resistance patterns, patient characteristics, and treatment practices may differ from those observed in other geographic regions. Therefore, caution should be exercised when extrapolating the findings to broader populations.

Third, molecular resistance testing was performed as part of routine clinical care in accredited external laboratories. Although all reported results were obtained from certified diagnostic facilities and standardized molecular methods were used, detailed information regarding assay performance characteristics, validation procedures, and laboratory-specific quality-control protocols was not consistently available for all patients. Consequently, methodological heterogeneity between laboratories cannot be completely excluded.

Fourth, the molecular analyses focused on the most frequently reported resistance-associated mutations within the *23S rRNA* and *gyrA* genes. Other genetic determinants of antimicrobial resistance, including fewer common mutations and resistance mechanisms involving additional genes, were not evaluated. Consequently, the overall burden of molecular resistance may have been underestimated.

Fifth, although information regarding the administered eradication treatment regimens was available and included in the multivariable analysis, treatment allocation was not randomized because of the retrospective study design. Consequently, residual confounding by indication cannot be completely excluded, and differences in treatment selection, patient adherence, treatment duration, or antibiotic exposure outside the documented medical history may have partially influenced the observed odds ratios and eradication outcomes.

Finally, although internal validation using bootstrap resampling demonstrated good model stability, external validation in independent patient cohorts was not performed. Consequently, the generalizability of the proposed prediction model remains uncertain and should be evaluated in future multicenter studies before routine clinical implementation can be recommended.

### 3.8. Future Directions

The findings of the present study provide several important directions for future research. First, prospective multicenter studies involving larger and more geographically diverse populations are needed to validate the associations observed between resistance-associated mutations, phenotypic antimicrobial resistance, and eradication outcomes. Such studies would help determine the generalizability of the present findings and provide a more comprehensive understanding of regional variations in molecular resistance patterns.

A major priority should be the external validation of the proposed prediction model. Although the model demonstrated excellent discriminatory performance within the present cohort, its reproducibility and clinical utility should be evaluated in independent patient populations before routine implementation can be recommended. Future studies should also investigate whether the incorporation of additional clinical, microbiological, and molecular variables could further improve predictive accuracy.

Further research should explore the integration of advanced molecular diagnostic techniques into routine clinical practice. The increasing availability of next-generation sequencing and multiplex molecular resistance panels may allow the simultaneous detection of multiple resistance determinants and provide a more comprehensive characterization of antimicrobial resistance profiles. Such approaches could facilitate more precise treatment selection and contribute to the development of personalized eradication strategies.

Another important area of investigation involves the evaluation of multidrug-resistant *H. pylori* strains. In the present study, patients harboring both *23S rRNA* and *gyrA* mutations experienced the poorest treatment outcomes. Future studies should further examine the epidemiology, clinical significance, and optimal management of dual-resistant and multidrug-resistant infections, which are likely to become increasingly relevant in the coming years.

The impact of molecular resistance testing on clinical decision-making and patient outcomes also warrants further investigation. Future prospective studies should assess whether routine implementation of molecular testing translates into improved eradication rates, fewer treatment failures, reduced antibiotic exposure, and lower healthcare costs when compared with empirical treatment strategies.

In addition, cost-effectiveness analyses are needed to determine the economic feasibility of routine molecular resistance testing in different healthcare settings. Understanding the balance between diagnostic costs and potential improvements in treatment success will be essential for supporting broader implementation of precision medicine approaches in gastroenterology.

Finally, future investigations should explore the development of integrated clinical decision-support systems combining molecular resistance markers, antimicrobial susceptibility profiles, patient characteristics, and predictive algorithms. Such tools may facilitate individualized treatment selection and contribute to optimizing the management of *H. pylori* infection in everyday clinical practice.

## 4. Materials and Methods

### 4.1. Study Design and Patient Selection

This retrospective real-world cohort study included consecutive adult patients diagnosed with *H. pylori* infection and evaluated at the Internal Medicine Department of the Oradea County Emergency Clinical Hospital, Oradea, Romania, between 1 November 2022 and 1 November 2025.

Clinical, endoscopic, histopathological, microbiological, molecular, and treatment outcome data were retrospectively collected from electronic medical records, paper-based medical files, endoscopy reports, microbiology laboratory records, and institutional databases.

Inclusion Criteria:

- Age ≥ 18 years;
- Confirmed *H. pylori* infection;
- Available antimicrobial susceptibility testing;
- Available molecular analysis of *23S rRNA* and *gyrA* mutations;
- Documented post-treatment eradication assessment;
- Complete clinical and endoscopic data.

Exclusion Criteria:

- Age < 18 years;
- Missing antimicrobial susceptibility testing;
- Missing molecular mutation analysis;
- Absence of post-treatment follow-up evaluation;
- Incomplete clinical records.

The final study cohort consisted of patients with complete clinical, endoscopic, microbiological, molecular, and treatment outcome data and was subsequently used for the development and validation of the prediction model for eradication failure.

### 4.2. Study Population

A total of 348 patients diagnosed with *H. pylori* infection were initially assessed for eligibility. Of these, 54 patients were excluded from the analysis: 19 due to missing antimicrobial susceptibility testing, 12 due to unavailable molecular mutation analysis, and 23 because post-treatment follow-up data were not available.

**Figure 5:**
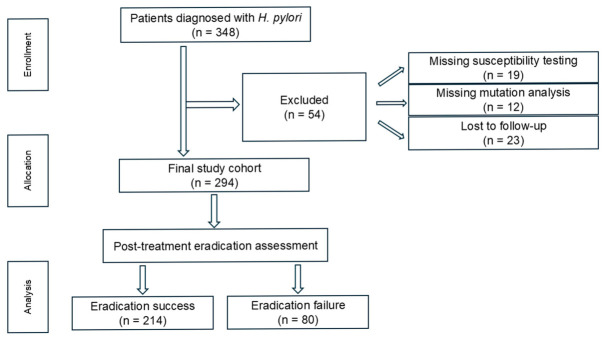
Flowchart of patient selection and study cohort formation.

The final study cohort included 294 patients with complete clinical, endoscopic, microbiological, molecular, and treatment outcome data. Eradication was successful in 214 patients (72.8%), whereas treatment failure was documented in 80 patients (27.2%). The patient selection process is illustrated in Figure 5.

### 4.3. Clinical, Endoscopic, and Histopathological Data Collection

Demographic and clinical data were retrospectively extracted from electronic medical records, paper-based medical files, endoscopy reports, and institutional databases. The collected variables included age, sex, BMI, smoking status, alcohol consumption, previous *H. pylori* eradication attempts, previous antibiotic exposure, eradication treatment regimen, presenting symptoms, and comorbidities. Treatment regimens were classified as clarithromycin-based triple therapy, bismuth quadruple therapy, levofloxacin-based therapy, or other regimens.

Presenting gastrointestinal symptoms, including dyspepsia, epigastric pain, bloating, and nausea, were recorded at the time of diagnosis. Comorbid conditions, including hypertension, dyslipidemia, diabetes mellitus, chronic liver disease, and family history of gastric cancer, were also documented.

All patients underwent upper gastrointestinal endoscopy as part of routine clinical evaluation. Endoscopic findings, including antral gastritis, pangastritis, erosive gastritis, gastric ulcer, and duodenal ulcer, were recorded from endoscopy reports.

Histopathological examination was performed on gastric biopsy specimens obtained during endoscopy according to routine clinical practice. Tissue sections were stained with hematoxylin–eosin and modified Giemsa stain (Merck, Darmstadt, Germany). Histological findings, including gastritis severity (mild, moderate, or severe), intestinal metaplasia, and gastric atrophy, were recorded and included in the analysis.

### 4.4. Diagnosis of H. pylori Infection

All patients underwent upper gastrointestinal endoscopy as part of routine clinical evaluation. Gastric biopsy specimens were obtained from the antrum and corpus according to standard endoscopic practice.

The diagnosis of *H. pylori* infection was established by histopathological examination and microbiological culture of gastric biopsy specimens. Histological assessment was performed by experienced pathologists, while bacterial isolation was carried out using Columbia Agar supplemented with 5% sheep blood (BD Diagnostics, Sparks, MD, USA) under microaerophilic conditions at 37 °C. Presumptive *H. pylori* colonies were identified based on colony morphology and confirmed by Gram staining and positive urease, catalase, and oxidase tests.

Only patients with confirmed *H. pylori* infection and available antimicrobial susceptibility testing and molecular resistance analysis were included in the study.

Eradication status was evaluated at least four weeks after completion of therapy using a ^13^C-urea breath test performed with the Heliprobe System (Kibion AB, Uppsala, Sweden) and/or stool antigen testing using the Premier Platinum HpSA assay (Meridian Bioscience, Cincinnati, OH, USA). Patients with negative post-treatment results were classified as having successful eradication, whereas those with persistent positive results were considered to have eradication failure.

### 4.5. Antimicrobial Susceptibility Testing

Antimicrobial susceptibility testing was performed on cultured *H. pylori* isolates obtained from gastric biopsy specimens. Following isolation, bacterial strains were cultured under microaerophilic conditions and tested for susceptibility to clarithromycin, levofloxacin, amoxicillin, metronidazole, tetracycline, and rifampicin.

Minimum inhibitory concentrations (MICs) were determined using the E-test method (bioMérieux, Marcy-l’Étoile, France) according to the manufacturer’s instructions. Antimicrobial susceptibility results were interpreted using the clinical breakpoints established by the European Committee on Antimicrobial Susceptibility Testing (EUCAST). The MIC resistance breakpoints were >0.5 mg/L for clarithromycin, >1 mg/L for levofloxacin, >0.125 mg/L for amoxicillin, >8 mg/L for metronidazole, >1 mg/L for tetracycline, and >1 mg/L for rifampicin. Isolates were classified as susceptible or resistant according to the corresponding EUCAST breakpoint values. No intermediate susceptibility category is defined by EUCAST for *H. pylori* clarithromycin or levofloxacin susceptibility testing. Phenotypic resistance profiles were subsequently compared with the presence of resistance-associated genetic mutations and treatment outcomes.

### 4.6. Molecular Detection of Resistance-Associated Mutations

Molecular resistance testing was performed in accredited private diagnostic laboratories in Oradea, Romania, as part of routine clinical management. Molecular test results were retrospectively retrieved from laboratory reports available in the patients’ medical records.

DNA was extracted from gastric biopsy specimens using the QIAamp DNA Mini Kit (Qiagen, Hilden, Germany). Detection of resistance-associated mutations was performed using real-time polymerase chain reaction (RT-PCR) on a Rotor-Gene Q platform (Qiagen, Hilden, Germany), according to the manufacturer’s instructions.

The molecular analyses targeted mutations associated with clarithromycin resistance within the *23S rRNA* gene, including the A2143G, A2142G, and A2142C substitutions. In addition, mutations associated with fluoroquinolone resistance were investigated within the *gyrA* gene, including the N87K, D91G, and D91N substitutions.

For the purposes of the present study, patients were classified according to the presence or absence of resistance-associated mutations. The detected molecular alterations were subsequently correlated with phenotypic antimicrobial susceptibility profiles, eradication outcomes, and treatment failure risk.

### 4.7. Eradication Treatment Protocols

Treatment regimens were selected according to contemporary international and national recommendations and were individualized based on antimicrobial susceptibility testing and molecular resistance profiles when available.

First-line treatment most commonly consisted of bismuth-containing quadruple therapy (proton pump inhibitor, bismuth, tetracycline, and metronidazole) administered for 14 days. In selected patients without evidence of clarithromycin resistance, clarithromycin-based triple therapy (proton pump inhibitor, amoxicillin, and clarithromycin) was prescribed for 14 days.

Patients who failed first-line treatment received second-line regimens based on antimicrobial susceptibility testing results and previous antibiotic exposure. Levofloxacin-containing therapies, bismuth-based quadruple regimens, or alternative susceptibility-guided treatments were used according to clinician judgment.

Because of the retrospective design of the study, complete information regarding the exact distribution of treatment regimens was not consistently available for all patients. Nevertheless, treatment selection followed contemporary guideline recommendations and local clinical practice.

### 4.8. Outcome Definition

The primary outcome of the study was *H. pylori* eradication failure following standard eradication therapy.

Eradication success was defined as a negative post-treatment ^13^C-urea breath test and/or stool antigen test performed at least four weeks after completion of treatment. Patients with negative follow-up testing were classified as having successful eradication.

Eradication failure was defined as persistent *H. pylori* infection confirmed by a positive post-treatment test result. Patients with positive follow-up testing were classified as having treatment failure.

For patients with multiple clinical visits during the study period, only the most recent eradication treatment episode with complete clinical, microbiological, molecular, treatment, and follow-up data was included in the analysis. Information regarding previous *H. pylori* eradication attempts was obtained from the documented medical history available at the time of the index treatment episode. Accordingly, the variable “previous eradication attempts” represented any documented eradication therapies administered before the treatment episode included in the present study.

For subsequent analyses, patients were stratified according to treatment outcome (eradication success versus eradication failure). This classification was used for comparative analyses, multivariable logistic regression, and development of the prediction model.

### 4.9. Statistical Analysis

Statistical analyses were performed using IBM SPSS Statistics software (version 26.0; IBM Corp., Armonk, NY, USA).

Continuous variables were expressed as mean ± standard deviation (SD), whereas categorical variables were presented as frequencies and percentages. Comparisons between groups were performed using the independent-samples Student’s *t*-test for continuous variables and the χ^2^ test or Fisher’s exact test, as appropriate, for categorical variables.

The association between resistance-associated mutations and phenotypic antimicrobial resistance was evaluated descriptively and graphically using heatmap visualization. Treatment outcomes were compared according to molecular profiles treatment regimens, and variables associated with eradication failure were initially assessed using univariate analyses.

Variables demonstrating statistical significance in univariate analysis (*p* < 0.05), together with treatment regimen as a clinically relevant covariate, were considered for inclusion in the multivariable logistic regression model to identify independent predictors of eradication failure. To minimize multicollinearity, overlapping variables representing the same biological construct were not entered simultaneously into the final model. Patients harboring concurrent *23S rRNA* and *gyrA* mutations remained in the analysis and contributed to both binary predictor variables. Because of the limited number of patients with dual mutations, interaction terms between *23S rRNA* and *gyrA* mutations were not included in the final model. aORs and corresponding 95% confidence intervals (95% CIs) were calculated.

A clinical prediction model for eradication failure was developed using the independent predictors identified in the multivariable analysis. Model discrimination was evaluated by ROC curve analysis and calculation of the AUC. Model calibration was assessed using the Hosmer–Lemeshow goodness-of-fit test. Internal validation of the prediction model was performed using bootstrap resampling with 1000 iterations. For each bootstrap sample, the logistic regression model was refitted and its performance was evaluated. Model optimism was estimated as the difference between apparent and bootstrap performance. Optimism-corrected estimates of discrimination and calibration were subsequently calculated. The calibration slope was used to assess model overfitting.

All statistical tests were two-sided, and a *p*-value < 0.05 was considered statistically significant.

### 4.10. Ethical Considerations

This retrospective cohort study was conducted in accordance with the ethical principles of the Declaration of Helsinki and applicable national regulations governing biomedical research involving human participants.

The study protocol was approved by the Research Ethics Subcommittee of the University of Oradea, Romania (Approval No. 86/31.03.2026), and by the Ethics Committee of the Oradea County Emergency Clinical Hospital, Oradea, Romania (Approval No. 33050/06.11.2025).

Due to the retrospective nature of the study and the use of anonymized clinical and laboratory data, the requirement for obtaining individual informed consent was waived by the ethics committees. Patient confidentiality was strictly maintained throughout the study, and all data were analyzed in anonymized form.

## 5. Conclusions

In conclusion, resistance-associated mutations within the *23S rRNA* and *gyrA* genes were frequently detected among patients with *H. pylori* infection and demonstrated strong associations with phenotypic clarithromycin and levofloxacin resistance. The A2143G mutation emerged as the predominant molecular determinant of clarithromycin resistance, while *gyrA* mutations were strongly associated with fluoroquinolone resistance. Patients harboring resistance-associated mutations, particularly those carrying mutations in both genes, experienced significantly lower eradication rates than patients infected with wild-type strains.

Furthermore, previous eradication attempts, A2143G mutation status, *gyrA* mutations, and increasing age were identified as independent predictors of eradication failure. A prediction model integrating molecular and clinical variables demonstrated excellent discriminatory performance and may represent a useful tool for risk stratification and individualized treatment selection.

Collectively, these findings highlight the clinical value of molecular resistance testing in the management of *H. pylori* infection and support the growing implementation of susceptibility-guided therapeutic strategies. Patients harboring concomitant *23S rRNA* and *gyrA* mutations represented the subgroup at highest risk of eradication failure and may particularly benefit from individualized treatment approaches. Future prospective multicenter studies are warranted to validate the proposed prediction model and further explore the role of molecular diagnostics in precision medicine approaches for *H. pylori* eradication.

## Figures and Tables

**Table 1:** Baseline Characteristics of the Study Population.

Variable	Total (n = 294)
**Demographic characteristics**	
Age (years), mean ± SD	53.6 ± 14.2
Female sex, n (%)	173 (58.8)
Urban residence, n (%)	211 (71.8)
BMI (kg/m^2^), mean ± SD	27.1 ± 4.8
**Lifestyle factors**	
Current smoker, n (%)	84 (28.6)
Alcohol consumption, n (%)	71 (24.1)
Previous medical history	
Previous eradication attempts, n (%)	81 (27.6)
Previous macrolide exposure, n (%)	69 (23.5)
Previous fluoroquinolone exposure, n (%)	52 (17.7)
PPI use before diagnosis, n (%)	202 (68.7)
**Presenting symptoms**	
Dyspepsia, n (%)	238 (81.0)
Epigastric pain, n (%)	188 (63.9)
Bloating, n (%)	125 (42.5)
Nausea, n (%)	67 (22.8)
**Comorbidities**	
Hypertension, n (%)	82 (27.9)
Dyslipidemia, n (%)	56 (19.0)
Diabetes mellitus, n (%)	34 (11.6)
Chronic liver disease, n (%)	18 (6.1)
Family history of gastric cancer, n (%)	21 (7.1)

**Table 2:** Endoscopic and Histopathological Findings.

Finding	n (%)
**Endoscopic findings**	
Antral gastritis	128 (43.5)
Pangastritis	62 (21.1)
Erosive gastritis	39 (13.3)
Gastric ulcer	28 (9.5)
Duodenal ulcer	17 (5.8)
**Histopathological findings**	
Mild gastritis	84 (28.6)
Moderate gastritis	141 (48.0)
Severe gastritis	36 (12.2)
Intestinal metaplasia	33 (11.2)
Gastric atrophy	21 (7.1)

**Table 3:** Distribution of *23S rRNA* and *gyrA* Mutations.

Variable	n (%)
***23S rRNA* mutations**	
A2143G	74 (25.2)
A2142G	19 (6.5)
A2142C	8 (2.7)
Any *23S rRNA* mutation	101 (34.4)
***gyrA* mutations**	
N87K	29 (9.9)
D91G	23 (7.8)
D91N	12 (4.1)
Any *gyrA* mutation	64 (21.8)
Both *23S rRNA* and *gyrA* mutations	27 (9.2)
Wild-type genotype	129 (43.9)

**Table 4:** Eradication Outcomes According to Mutation Status.

Mutation Status	Success n (%)	Failure n (%)	*p*-Value
Wild type (n = 129)	117 (90.7)	12 (9.3)	Reference
Any *23S rRNA* mutation (n = 101)	60 (59.4)	41 (40.6)	<0.001
A2143G (n = 74)	43 (58.1)	31 (41.9)	<0.001
A2142G (n = 19)	12 (63.2)	7 (36.8)	0.002
A2142C (n = 8)	5 (62.5)	3 (37.5)	0.041
N87K (n = 29)	18 (62.1)	11 (37.9)	0.003
D91G (n = 23)	13 (56.5)	10 (43.5)	0.001
D91N (n = 12)	8 (66.7)	4 (33.3)	0.027
Any *gyrA* mutation (n = 64)	39 (61.4)	25 (38.6)	<0.001
Both *23S rRNA* and *gyrA* mutations (n = 27)	12 (44.4)	15 (55.6)	<0.001

Note: *p*-values were calculated using Pearson’s χ^2^ test or Fisher’s exact test, as appropriate. Each mutation category was compared with the wild-type group. No adjustment for multiple comparisons was applied because these analyses were exploratory.

**Table 5:** Distribution of Eradication Treatment Regimens According to *H. pylori* Eradication Outcome.

Treatment Regimen	Success (n = 214)	Failure (n = 80)	*p*-Value
Clarithromycin triple therapy (n = 162)	106 (65.4%)	56 (34.6%)	<0.001
Bismuth quadruple therapy (n = 78)	71 (91.0%)	7 (9.0%)	Reference
Levofloxacin-based therapy (n = 41)	28 (68.3%)	13 (31.7%)	0.018
Other regimens (n = 13)	9 (69.2%)	4 (30.8%)	0.221

Note: Bismuth quadruple therapy was used as the reference category for comparisons of eradication outcomes.

**Table 6:** Characteristics of Patients According to Treatment Outcome.

Variable	Success (n = 214)	Failure (n = 80)	*p*-Value
Age (years), mean ± SD	51.9 ± 13.4	58.2 ± 14.1	0.004
Female sex, n (%)	126 (58.9)	47 (58.8)	0.987
Urban residence, n (%)	150 (70.1)	61 (76.3)	0.287
BMI (kg/m^2^), mean ± SD	26.9 ± 4.7	27.8 ± 5.0	0.148
Current smoker, n (%)	58 (27.1)	26 (32.5)	0.372
Previous eradication attempts, n (%)	39 (18.2)	41 (51.3)	<0.001
Previous macrolide exposure, n (%)	38 (17.8)	31 (38.8)	<0.001
Previous fluoroquinolone exposure, n (%)	24 (11.2)	28 (35.0)	<0.001
Clarithromycin resistance, n (%)	49 (22.9)	49 (61.3)	<0.001
Levofloxacin resistance, n (%)	36 (16.8)	33 (41.3)	<0.001
Any *gyrA* mutation, n (%)	34 (15.9)	30 (37.5)	<0.001
A2143G mutation, n (%)	41 (19.2)	42 (52.5)	<0.001
Both *23S rRNA* and *gyrA* mutations, n (%)	11 (5.1)	16 (20.0)	<0.001

Note: *p*-values were calculated using the independent-samples Student’s *t*-test for continuous variables and Pearson’s χ^2^ test or Fisher’s exact test, as appropriate, for categorical variables. No adjustment for multiple comparisons was applied because these analyses were exploratory.

**Table 7:** Multivariable Logistic Regression Analysis of Factors Associated with Eradication Failure.

Variable	Crude OR (95% CI)	*p*-Value	Adjusted OR (95% CI)	*p*-Value
Age (per year increase)	1.04 (1.02–1.06)	0.002	1.03 (1.01–1.05)	0.015
Previous eradication attempts	4.73 (2.71–8.26)	<0.001	3.12 (1.71–5.68)	<0.001
A2143G mutation	4.91 (2.82–8.54)	<0.001	4.86 (2.43–9.72)	<0.001
Any *gyrA* mutation	3.17 (1.82–5.51)	<0.001	2.91 (1.45–5.84)	0.003
Clarithromycin-based triple therapy	3.68 (1.82–7.42)	<0.001	2.18 (1.02–4.63)	0.043
Bismuth quadruple therapy	Reference	—	Reference	—

**Table 8:** Clinical Risk Score for Prediction of Eradication Failure.

Predictor	Points
Age > 60 years	1
Previous eradication attempt	2
*gyrA* mutation	2
A2143G mutation	3
Clarithromycin-based triple therapy	1

**Table 9:** Risk Stratification According to the Clinical Prediction Score.

Total Score	Risk Category	Failure Rate
0–2	Low risk	7.2%
3–5	Intermediate risk	26.4%
≥6	High risk	68.1%

## Data Availability

The data presented in this study are available from the corresponding authors upon reasonable request. The data are not publicly available due to privacy and ethical restrictions related to patient confidentiality.

## Abbreviations

The following abbreviations are used in this manuscript:

aOR	Adjusted Odds Ratios
AUC	The Area Under the Curve
BMI	Body Mass Index
CI	Confidence Interval
EUCAST	European Committee on Antimicrobial Susceptibility Testing
*H. pylori*	*Helicobacter pylori*
MIC	Minimum Inhibitory Concentration
ROC	Receiver Operating Characteristic
SD	Standard Deviation

## References

[1] Chivu R.F., Bobirca F., Melesteu I., Patrascu T. (2024). The role of *Helicobacter pylori* infection in the development of gastric cancer—Review of the literature. Chirurgia.

[2] Park J.Y., Lee Y.C., Moayyedi P., Lansdorp-Vogelaar I., Camargo M.C., Tepeš B., Abnet C.C., Choi I.J., Clifford G., Dinis-Ribeiro M. (2026). *Helicobacter pylori* screen-and-treat programs for gastric cancer prevention—IARC Working Group report. N. Engl. J. Med..

[3] Li Y., Choi H., Leung K., Jiang F., Graham D.Y., Leung W.K. (2023). Global prevalence of *Helicobacter pylori* infection between 1980 and 2022: A systematic review and meta-analysis. Lancet Gastroenterol. Hepatol..

[4] Chen Y.C., Malfertheiner P., Yu H.T., Kuo C.L., Chang Y.Y., Meng F.T., Wu Y.X., Hsiao J.L., Chen M.J., Lin K.P. (2024). Global prevalence of *Helicobacter pylori* infection and incidence of gastric cancer between 1980 and 2022. Gastroenterology.

[5] Rocha G.R., Lemos F.F.B., Silva L.G.O., Luz M.S., Correa Santos G.L., Rocha Pinheiro S.L., Calmon M.S., de Melo F.F. (2025). Overcoming antibiotic-resistant *Helicobacter pylori* infection: Current challenges and emerging approaches. World J. Gastroenterol..

[6] Malfertheiner P., Megraud F., Rokkas T., Gisbert J.P., Liou J.M., Schulz C., Gasbarrini A., Hunt R.H., Leja M., O’Morain C. (2022). Management of *Helicobacter pylori* infection: The Maastricht VI/Florence consensus report. Gut.

[7] Casey O., Dobric M., Kelly O., O’Morain C.A. (2026). The importance of *Helicobacter pylori* eradication: A narrative review. Front. Gastroenterol..

[8] Mathew J. (2020). *H. pylori* eradication therapy reduces gastric cancer in patients with or without gastric neoplasia. Ann. Intern. Med..

[9] Ho J.J.C., Navarro M., Sawyer K., Elfanagely Y., Moss S.F. (2022). *Helicobacter pylori* antibiotic resistance in the United States between 2011 and 2021: A systematic review and meta-analysis. Am. J. Gastroenterol..

[10] Yu Y., Xue J., Lin F., Liu D., Zhang W., Ru S., Jiang F. (2024). Global primary antibiotic resistance rate of *Helicobacter pylori* in recent 10 years: A systematic review and meta-analysis. Helicobacter.

[11] Homan M., Jones N.L., Bontems P., Carroll M.W., Czinn S.J., Gold B.D., Goodman K., Harris P.R., Jerris R., Kalach N. (2024). Updated joint ESPGHAN/NASPGHAN guidelines for management of *Helicobacter pylori* infection in children and adolescents (2023). J. Pediatr. Gastroenterol. Nutr..

[12] Sharara A.I., Alsohaibani F.I., Alsaegh A., Al Ejji K., Al Awadhi S., Malfertheiner P., Karam S.A., Al-Taweel T. (2025). First regional consensus on the management of *Helicobacter pylori* infection in the Middle East. World J. Gastroenterol..

[13] Deane C., Kelly O., O’Morain C. (2024). Current and future perspectives on the management of *Helicobacter pylori*: A narrative review. Antibiotics.

[14] Park G., Kim B., Chung H., Kim S.G., Cho S.J. (2023). Types of 23S ribosomal RNA point mutations affecting *Helicobacter pylori* eradication rates in clarithromycin-based triple therapy. Korean J. Helicobacter Up. Gastrointest. Res..

[15] Albasha A.M., Elnosh M.M., Osman E.H., Zeinalabdin D.M., Fadl A.A.M., Ali M.A., Altayb H.N. (2021). *Helicobacter pylori* 23S rRNA gene A2142G, A2143G, T2182C, and C2195T mutations associated with clarithromycin resistance detected in Sudanese patients. BMC Microbiol..

[16] Chey W.D., Howden C.W., Moss S.F., Morgan D.R., Greer K.B., Grover S., Shah S.C. (2024). ACG clinical guideline: Treatment of *Helicobacter pylori* infection. Am. J. Gastroenterol..

[17] Šamanić I., Dadić B., Sanader Maršić Ž., Dželalija M., Maravić A., Kalinić H., Vrebalov Cindro P., Šundov Ž., Tonkić M., Tonkić A. (2023). Molecular characterization and mutational analysis of clarithromycin- and levofloxacin-resistance genes in *Helicobacter pylori* from gastric biopsies in Southern Croatia. Int. J. Mol. Sci..

[18] Medakina I., Tsapkova L., Polyakova V., Nikolaev S., Yanova T., Dekhnich N., Khatkov I., Bordin D., Bodunova N. (2023). *Helicobacter pylori* antibiotic resistance: Molecular basis and diagnostic methods. Int. J. Mol. Sci..

[19] Tran V.H., Nguyen T.M.N., Le P.T.Q., Nguyen T.H.T., Nguyen T.C.L., Ha T.M.T. (2024). Current status of *Helicobacter pylori* resistance to clarithromycin and levofloxacin in Vietnam: Results from molecular analysis of gastric biopsy specimens. J. Glob. Antimicrob. Resist..

[20] Paul S., Karmakar B.C., Roy N., Chaudhuri S., Mukhopadhyay A.K. (2025). Analysis of antimicrobial resistance patterns and genetic mutations in *Helicobacter pylori* from West Bengal, India depicting escalating clarithromycin and high levofloxacin resistance. Gut Pathog..

[21] Kim S.Y., Park J.M., Lim C.H., Lee H.A., Shin G.Y., Choe Y., Cho Y.K., Choi M.G. (2021). Types of 23S ribosomal RNA point mutations and therapeutic outcomes for *Helicobacter pylori*. Gut Liver.

[22] Marques A.T., Vítor J.M.B., Santos A., Oleastro M., Vale F.F. (2020). Trends in *Helicobacter pylori* resistance to clarithromycin: From phenotypic to genomic approaches. Microb. Genom..

[23] Kim I., Shin Y.R., Kim J.S., Kim B.W., Maeng L.S., Kim J.M. (2024). Detection of clarithromycin resistance in *Helicobacter pylori* using MmaxSure™ H. pylori & ClaR assay. Dig. Dis..

[24] Hasanuzzaman M., Bang C.S., Gong E.J. (2024). Antibiotic resistance of *Helicobacter pylori*: Mechanisms and clinical implications. J. Korean Med. Sci..

[25] Bodunova N., Tsapkova L., Polyakova V., Baratova I., Rumyantsev K., Dekhnich N., Nikolskaya K., Chebotareva M., Voynovan I., Parfenchikova E. (2024). Genetic markers of *Helicobacter pylori* resistance to clarithromycin and levofloxacin in Moscow, Russia. Curr. Issues Mol. Biol..

[26] Starkova D., Gladyshev N., Polev D., Saitova A., Egorova S., Svarval A. (2024). First insight into the whole genome sequence variations in clarithromycin-resistant *Helicobacter pylori* clinical isolates in Russia. Sci. Rep..

[27] Serena P., Mare R., Miutescu B., Bende R., Popa A., Aragona G., Seclăman E., Serena L., Barbulescu A., Sirli R. (2025). Resistance to clarithromycin and fluoroquinolones in *Helicobacter pylori* isolates: A prospective molecular analysis in Western Romania. Antibiotics.

[28] Xue Z., Zhao Q., Pei F., Gong Y., Wang F., Wang Y., Chen Q., Li Y., Xu Q., Tian J. (2025). *Helicobacter pylori* antimicrobial resistance and gene variants in Shandong Province. Sci. Rep..

[29] Hussein R.A., Al-Ouqaili M.T.S., Majeed Y.H. (2022). Detection of clarithromycin resistance and 23S rRNA point mutations in clinical isolates of *Helicobacter pylori*: Phenotypic and molecular methods. Saudi J. Biol. Sci..

[30] Haumaier F., Schneider-Fuchs A., Backert S., Vieth M., Sterlacci W., Wöhrl B.M. (2022). Rapid detection of quinolone resistance mutations in gyrA of *Helicobacter pylori* by real-time PCR. Pathogens.

[31] Álvarez-Aldana A., Beltrán-Angarita L., Guaca-González Y.M., Velandia-López M.A., Boyanova L. (2026). Levofloxacin and rifampin resistance in *Helicobacter pylori* isolates from Central-Western Colombia: Role of gyrA mutations in fluoroquinolone resistance. Antibiotics.

[32] Rhie S.Y., Park J.Y., Shin T.-S., Kim J.W., Kim B.J., Kim J.G. (2020). Discovery of a novel mutation in DNA gyrase and changes in the fluoroquinolone resistance of *Helicobacter pylori* over a 14-year period: A single-center study in Korea. Antibiotics.

[33] González-Hormazábal P., Arenas A., Serrano C., Pizarro M., Fuentes-López E., Arnold J., Berger Z., Musleh M., Valladares H., Lanzarini E. (2021). Prevalence of *Helicobacter pylori* antimicrobial resistance among Chilean patients. Arch. Med. Res..

[34] Fauzia K.A., Aftab H., Tshibangu-Kabamba E., Alfaray R.I., Saruuljavkhlan B., Cimuanga-Mukanya A., Matsumoto T., Subsomwong P., Akada J., Miftahussurur M. (2023). Mutations related to antibiotics resistance in *Helicobacter pylori* clinical isolates from Bangladesh. Antibiotics.

[35] Binmaeil H., Hanafiah A., Mohamed Rose I., Raja Ali R.A. (2021). Development and validation of multiplex quantitative PCR assay for detection of *Helicobacter pylori* and mutations conferring resistance to clarithromycin and levofloxacin in gastric biopsy. Infect. Drug Resist..

[36] Xu Y., Hao J.W., Min C.C., Yang L., Ma C.P., Shi C., Mao T., Tian Z.B., Wang T., Yu Y.N. (2025). Precision therapy guided by genotypic antibiotic resistance for *Helicobacter pylori* eradication: A prospective, randomized controlled trial. World J. Gastroenterol..

[37] Dascălu R.I., Bolocan A., Păduaru D.N., Constantinescu A., Mitache M.M., Stoica A.D., Andronic O. (2023). Multidrug resistance in *Helicobacter pylori* infection. Front. Microbiol..

[38] Mannion A., Dzink-Fox J., Shen Z., Piazuelo M.B., Wilson K.T., Correa P., Peek R.M., Camargo M.C., Fox J.G. (2021). *Helicobacter pylori* antimicrobial resistance and gene variants in high- and low-gastric-cancer-risk populations. J. Clin. Microbiol..

[39] Lok C.H., Zhu D., Wang J., Ren Y.T., Jiang X., Li S.J., Zhao X.Y. (2020). Phenotype and molecular detection of clarithromycin and levofloxacin resistance in *Helicobacter pylori* clinical isolates in Beijing. Infect. Drug Resist..

[40] Zhang Y., Wen Y., Xiao Q., Zheng W., Long G., Chen B., Shu X., Jiang M. (2020). Mutations in the antibiotic target genes related to clarithromycin, metronidazole and levofloxacin resistance in *Helicobacter pylori* strains from children in China. Infect. Drug Resist..

[41] Losurdo G., Giorgio F., Pricci M., Girardi B., Russo F., Riezzo G., Martulli M., Piazzolla M., Cocomazzi F., Abbruzzi F. (2020). *Helicobacter pylori* primary and secondary genotypic resistance to clarithromycin and levofloxacin detection in stools: A 4-year scenario in Southern Italy. Antibiotics.

[42] Wang P., Zhao J., Lv B., Zhang X., Li M., Chen S., Fan B., Wang W. (2026). Genomic insights into antibiotic resistance, virulence traits and phylogenetic lineages of 141 clinical *Helicobacter pylori* isolates from Eastern China. Front. Cell. Infect. Microbiol..

[43] Van den Poel B., Gils S., Micalessi I., Carton S., Christiaens P., Cuyle P.J., Moons V., Van Olmen G., Smismans A., Bourgain C. (2021). Molecular detection of *Helicobacter pylori* and clarithromycin resistance in gastric biopsies: A prospective evaluation of RIDA^®^GENE *Helicobacter pylori* assay. Acta Clin. Belg..

[44] Pichon M., Pichard B., Barrioz T., Plouzeau C., Croquet V., Fotsing G., Chéron A., Vuillemin É., Wangermez M., Haineaux P.A. (2020). Diagnostic accuracy of a noninvasive test for detection of *Helicobacter pylori* and resistance to clarithromycin in stool by the Amplidiag H. pylori+ClariR real-time PCR assay. J. Clin. Microbiol..

[45] Osser B., Toth C., Nistor-Cseppento C.D., Ilia I., Osser G., Cevei M., Aur C., Fazakas R., Bondar L.I. (2025). Effects of structured physical therapy on spinal alignment in idiopathic scoliosis: A 12-month prospective study. Diagnostics.

[46] Kebotsamang T., Munkombwe D., Bwalya L., Kelly P., Kayamba V. (2024). Prevalence of clarithromycin-resistant *Helicobacter pylori* strains in Zambia: A Sub-Saharan African country. Dig. Dis..

[47] Cho S.H., Park M.S., Park S.Y., Kim D.H., You H.S., Kim H.S. (2023). Effectiveness of 7-day triple therapy with half-dose clarithromycin for the eradication of *Helicobacter pylori* without the A2143G and A2142G point mutations of the 23S rRNA gene in a high clarithromycin resistance area. Front. Med..

[48] Yuan G.L., Yang J., Peng Y., Lai J.X., Li Z.K. (2025). Current status of *Helicobacter pylori* infection and prevalence of resistance-associated gene mutations in Bortala Mongolian Autonomous Prefecture, Xinjiang: A single-center study. Front. Microbiol..

[49] Salahi-Niri A., Nabavi-Rad A., Monaghan T.M., Rokkas T., Doulberis M., Sadeghi A., Zali M.R., Yamaoka Y., Tacconelli E., Yadegar A. (2024). Global prevalence of *Helicobacter pylori* antibiotic resistance among children in the World Health Organization regions between 2000 and 2023: A systematic review and meta-analysis. BMC Med..

[50] Li C.L., Zhou K., Zhang Y.X., Suo B.J., Tian X.L., Zhang Y.X., Ren X.L., Shi Y.Y., Zhou L.Y., Song Z.Q. (2024). Tailored therapy guided by genotypic resistance of clarithromycin and levofloxacin detected by polymerase chain reaction in the first-line treatment of *Helicobacter pylori* infection. J. Dig. Dis..

[51] Contreras M., Mujica H., García-Amado M.A. (2024). Molecular tools of antibiotic resistance for *Helicobacter pylori*: An overview in Latin America. Front. Gastroenterol..

[52] Cortés P., Nelson A.D., Bi Y., Stancampiano F.F., Murray L.P., Pujalte G.G.A., Gomez V., Harris D.M. (2021). Treatment approach of refractory *Helicobacter pylori* infection: A comprehensive review. J. Prim. Care Community Health.

[53] Kocsmár É., Buzás G.M., Szirtes I., Kocsmár I., Kramer Z., Szijártó A., Fadgyas-Freyler P., Szénás K., Rugge M., Fassan M. (2021). Primary and secondary clarithromycin resistance in *Helicobacter pylori* and mathematical modeling of the role of macrolides. Nat. Commun..

[54] Nestegard O., Moayeri B., Halvorsen F.A., Tønnesen T., Sørbye S.W., Paulssen E., Johnsen K.M., Goll R., Florholmen J.R., Melby K.K. (2022). *Helicobacter pylori* resistance to antibiotics before and after treatment: Incidence of eradication failure. PLoS ONE.

[55] Spagnuolo R., Scarlata G.G.M., Paravati M.R., Abenavoli L., Luzza F. (2024). Change in diagnosis of *Helicobacter pylori* infection in the treatment-failure era. Antibiotics.

[56] Fazakas R., Bondar L.I., Toth C., Miuța C.C., Ilia I., Toderescu C.D., Pop A. (2025). Temporal patterns and treatment associations in complications following hip arthroplasty. Diagnostics.

[57] Cho J.H., Jin S.Y. (2022). Current guidelines for *Helicobacter pylori* treatment in East Asia 2022: Differences among China, Japan, and South Korea. World J. Clin. Cases.

[58] Bondar L.I., Iovanovici D.C., Măduța V., Butari D.B., Șandor F.M., Mariș M.A., Piroș L.E., Miuța C.C., Toderescu C.D., Popescu M.I. (2025). Screening depression in ischemic heart disease: Gender differences and psychosocial implications using a self-developed questionnaire. J. Clin. Med..

[59] Schulz C., Liou J.M., Alboraie M., Bornschein J., Campos Nunez C., Coelho L.G., Quach D.T., Fallone C.A., Chen Y.C., Gerhard M. (2025). *Helicobacter pylori* antibiotic resistance: A global challenge in search of solutions. Gut.

[60] Narasimhan V., Pulakkat Warrier S., John J.J., T. M.P., Varadaraj N., Thomas G.G., Veeraraghavan B. (2026). Predicting clinical outcomes in *Helicobacter pylori*-positive patients using supervised learning through the integration of demographic and genomic features. BMC Gastroenterol..

[61] Moss S.F., Shah S.C., Tan M.C., El-Serag H.B. (2024). Evolving concepts in *Helicobacter pylori* management. Gastroenterology.

[62] Lin Y., Shao Y., Yan J., Ye G. (2023). Antibiotic resistance in *Helicobacter pylori*: From potential biomolecular mechanisms to clinical practice. J. Clin. Lab. Anal..

[63] Osser G., Osser B., Toth C., Miuța C.C., Marconi G.R., Bondar L.I. (2024). Exploring the relationship between ejection fraction, arterial stiffness, NT-proBNP, and hospitalization risk in heart failure patients. Diagnostics.

[64] Marascio N., Scarlata G.G.M., Romeo F., Cicino C., Trecarichi E.M., Quirino A., Torti C., Matera G., Russo A. (2023). The Role of Gut Microbiota in the Clinical Outcome of Septic Patients: State of the Art and Future Perspectives. Int. J. Mol. Sci..

[65] Joshi G., Rani S., Bharti D., Panda N., Chavan P., Mathpal S., Ramaiah S., Anbarasu A. (2026). The role of the gut microbiome in antibiotic-driven antimicrobial resistance. Front Microbiol..

